# Area-Level Social Determinants of Alcohol-Related Mortality: Knowledge Gaps and Implications for Community Health

**DOI:** 10.35946/arcr.v44.1.06

**Published:** 2024-10-31

**Authors:** Katherine J. Karriker-Jaffe, La Sonya A. Goode, Shannon M. Blakey, Jamie L. Humphrey, Pamela A. Williams, Ivette Rodriguez Borja, Jessica Cance, Georgiy Bobashev

**Affiliations:** 1 RTI International, Oakland, California; 2 RTI International, Research Triangle Park, North Carolina

**Keywords:** alcohol, alcohol-related disorders, mortality, social determinants of health, socioeconomic factors, social context, community health

## Abstract

**PURPOSE:**

Rates of alcohol-related mortality (including deaths attributed to chronic alcohol use as well as acute causes involving alcohol) have been increasing in the United States, particularly for certain population subgroups, such as women. This review summarizes associations of area-level social determinants of health with alcohol-related mortality. These determinants, measured at the community, county, or state level, include alcohol control policies, health care availability, and a community’s socioeconomic environment. Examining multiple geographic levels illuminates how macro-level social determinants and local contexts contribute to alcohol-related mortality to inform intervention. Attention to the broad variety of social determinants of alcohol-related mortality could ultimately improve community health.

**SEARCH METHODS:**

A literature search of three databases—PubMed, Web of Science, and Cumulative Index to Nursing and Allied Health Literature (CINAHL)—conducted between March 13 and May 16, 2023, identified peer-reviewed studies published from 1990 to May 2023 that modeled at least one area-level social determinant of health as a predictor or correlate of area-level rates of alcohol-related mortality in the United States. Unpublished dissertations, commentaries, editorials, review papers, and articles published in languages other than English were excluded. Two team members reviewed each abstract to verify that the article addressed alcohol-related mortality and included at least one area-level social determinant of health.

**SEARCH RESULTS:**

The authors screened 313 abstracts and excluded 210 that did not meet inclusion criteria. The full texts of 103 articles were retrieved. Upon further screening, 30 articles were excluded (two were not obtained), leaving 71 studies for detailed review.

**DISCUSSION AND CONCLUSIONS:**

Many studies analyzed fatal alcohol-related motor vehicle crashes or cirrhosis/liver disease mortality. Fewer analyzed other mortality causes related to chronic alcohol consumption. No studies focused on racism and discrimination, community-level prevention activities, or community social services in relation to alcohol-related mortality. Few studies examined major health policy changes or addressed health care system factors. Although the variation across studies complicates systematic comparison of the results, some key themes did emerge from the reviewed studies, such as the beneficial effects of stronger alcohol policies and the importance of socioeconomic conditions as determinants of alcohol-related mortality. Research using a more diverse set of theoretically informed social determinants may help examine whether, how, and for whom racism and discrimination as well as health policies and social services impact alcohol-related mortality. Finally, there is a gap in research linking local community contexts with alcohol-related mortality. Better understanding of subgroup differences, interactions between different contextual factors, and specific mechanisms of action may help identify promising new strategies to improve population health and reduce alcohol-related mortality.

More than 50 causes of death are linked to excessive alcohol use.^1^ This broad range of conditions—including those caused by chronic alcohol use, such as cirrhosis or cancers, as well as by acute events associated with heavy episodic or binge alcohol use, such as injuries—results in more than 178,000 annual deaths in the United States^2^ and more than 3 million annual deaths globally.^3^ In one U.S. study, excessive alcohol use (e.g., daily alcohol consumption of more than 2 drinks for women and more than 4 drinks for men) was estimated to contribute to 12.9% of the mortality of the population between ages 20 to 69, with large differences by sex/gender (15% in men, 9.4% in women).^1^ Additionally, there was wide variability between U.S. states, with estimates ranging from 9.3% of total deaths in Mississippi to 21.7% in New Mexico attributable at least partly to alcohol.^1^ Results from a meta-analysis suggest that people with alcohol use disorder (AUD) have higher mortality risk compared to the general population as well as to people without AUD who drink heavily.^4^ Mortality trend data show that alcohol-related deaths increased markedly between 2013 and 2016 across sex/gender and racial and ethnic groups,^5^ and these deaths continued to increase between 2019 and 2020.^6^ This review uses a social determinants of health (SDOH) framework to broadly conceptualize area-level characteristics that may influence alcohol-related mortality.

Alcohol-related mortality includes deaths caused by acute individual behaviors, such as driving under the influence (DUI), and deaths caused by chronic heavy alcohol consumption.^1^ Many studies have focused on the contributions of chronic and acute alcohol use and AUD to specific causes of mortality and on variability in alcohol-related mortality across demographic and geographic subgroups. SDOH also contribute to alcohol-related deaths^7^ and may help explain demographic and geographic variations in mortality. As conceptualized by the U.S. Department of Health and Human Services^8^ and the World Health Organization,^9^ SDOH encompass a broad range of social, economic, and political conditions present in the environments where people live, work, and relax, including social integration, exposure to racial or other forms of discrimination, educational and economic conditions, and access to high-quality health care and social services.^8^,^9^

This review focuses on area-level SDOH likely to be linked to mortality resulting from either acute or chronic alcohol-related causes. For alcohol-related deaths attributable to acute intoxication (e.g., those caused by motor vehicle crashes [MVCs] or violence), social and policy factors related to the promotion and control of excessive alcohol consumption are especially relevant.^7^ Structural factors in the built environment, such as roadway design and lighting, also may play a role, particularly in rural areas. Alcohol availability and alcohol control policies, along with health care availability, also are germane for deaths attributable to alcohol misuse or AUD. Adequate health care is crucial for treating chronic physical health conditions caused or exacerbated by alcohol use and behavioral health conditions such as AUD, depression, and anxiety. As described by Monnat,^10^ socioeconomic disadvantages are likely determinants of higher drug-related (and alcohol-related) mortality through effects of economic stressors on family relationships, social connections, hopelessness, and social disorder. Conversely, indicators of social capital (such as community engagement and social cohesion) may serve to buffer against social isolation and depression, resulting in lower drug- and alcohol-related mortality. Socioecological frameworks of human development have identified key contextual factors (i.e., SDOH) at the state, county, and community levels that are hypothesized to be related to mortality from chronic and/or acute alcohol consumption and that provide a guiding taxonomy for this review (see Figure 1).^11^ Examining evidence across these multiple levels of influence enables understanding of how macro and more local social determinants contribute to alcohol-related mortality, which can help inform intervention.

Recent reviews have explored social determinants of opioid and other drug overdose mortality.^12^–^14^ However, such studies often overlook alcohol-related mortality, even though alcohol is the most commonly co-used substance among people who misuse opioids.^15^ There also are many shared causes contributing to recent mortality trends related to alcohol and other drug use, such as the “deaths of despair” theory, which centers on deaths involving drug and/or alcohol overdose, alcohol-related diseases, and suicide.^16^–^18^ Social distress has been identified as an upstream explanatory factor related to overdose mortality and deaths of despair, but strong gender differences suggest that causes of death might not share the same underlying factors for men and women.^17^,^18^

A recent systematic review of factors associated with drug overdose mortality in the United States^14^ identified consistent associations with greater economic strain, mining employment (compared to other sectors), less substance use disorder treatment availability, less social capital, and greater density of marijuana dispensaries. A small scoping review of social determinants of deaths of despair in the United States^19^ found associations with rurality, low socioeconomic position, high job insecurity, and high unemployment. The systematic review also found that associations of health care professional shortages, physicians per capita, and socioeconomic context with overdose mortality often differed for groups defined by race/ethnicity, sex/gender, age, and rurality.^14^ Specific contextual factors affecting excessive alcohol use and subsequent mortality likely also vary across population subgroups.

To identify actionable policy and intervention opportunities, this review examines extant literature on area-level SDOH associated with alcohol-related mortality. By highlighting robust evidence and identifying knowledge gaps, the review aims to provide insights for evidence-based population-level strategies to reduce alcohol-related mortality and promote healthier communities.

## Search Methods Employed

Between March 13 and May 16, 2023, the authors searched PubMed, Web of Science, and the Cumulative Index to Nursing and Allied Health Literature (CINAHL) for peer-reviewed studies published from January 1990 to May 2023 that modeled at least one area-level indicator (e.g., county poverty rates, alcohol outlet density, alcohol policies, racism/discrimination, alcohol treatment access) as a predictor or correlate of area-level rates of alcohol-related mortality. Outcomes of interest were alcohol-related deaths due to either chronic or acute alcohol consumption, including those causes that were 100% alcohol-attributable (such as alcohol-associated liver disease) as well as those only partially attributable to alcohol (such as cancers).^1^ Accordingly, the search strategy for this review included broad, cause-unspecified terms (e.g., “alcohol*” AND (“mortality” OR “death” OR “fatal*”)) as well as cause-specific terms (e.g., cirrhosis). Table 1 presents a detailed list of search terms for each database. This review also includes studies where alcohol-related mortalities were combined with drug-related mortalities in a single outcome (e.g., “drug- and/or alcohol-related mortality”). U.S. studies using general population and subgroup data are included. Excluded from this review were unpublished dissertations, commentaries or editorials, literature reviews, and articles published in languages other than English.

Review methods focused on identifying how studies approached area-level SDOH and different mortality outcomes. After removing duplicates, two reviewers screened each abstract to verify that the article examined the association between alcohol-related mortality and at least one area-level variable. The authors reviewed the full texts when the abstract lacked sufficient information to determine eligibility. Also reviewed were reference lists of included papers and relevant review articles to identify additional cited references that met inclusion criteria. A limitation of the search strategy is that, although approximately half of cirrhosis and liver disease deaths are related to alcohol,^20^ not all studies specified whether they included only alcohol-related cirrhosis/liver disease or any type of cirrhosis/liver disease. Thus, this review may include mortality from liver diseases not attributable to alcohol. Another limitation is the systematic exclusion of general mortality outcomes and deaths that might be related to alcohol (e.g., homicide) as well as other serious alcohol-related outcomes (e.g., alcohol-involved MVCs or violence) that did not result in mortality.

## Search Results

**Figure 2:**
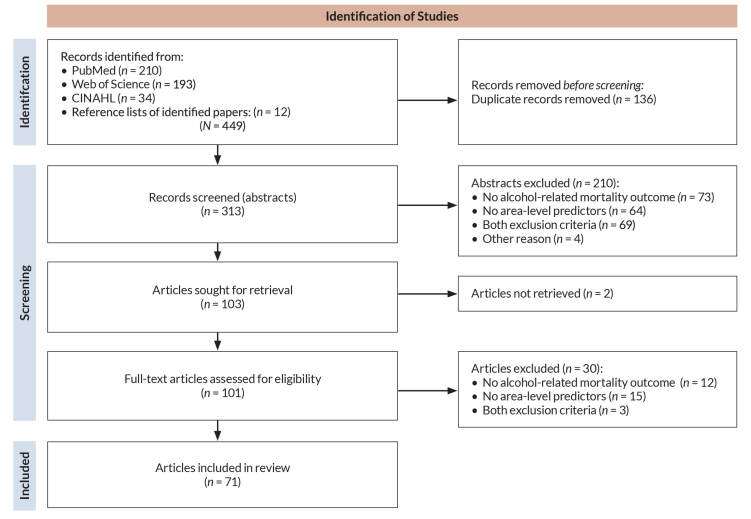
Flow diagram for study selection during the narrative review of area-level social determinants of alcohol-related mortality. *Note:* CINAHL, Cumulative Index to Nursing and Allied Health Literature.

The database searches yielded 437 total records (210 from PubMed, 193 from Web of Science, 34 from CINAHL). Another 12 articles were identified from reference lists. After removing 136 duplicate records, 313 unique abstracts remained for screening. Of these, 210 abstracts were excluded that did not meet inclusion criteria, such as not examining alcohol-related mortality (*n* = 142), not including area-level variables (*n* = 64), or not being U.S.-based studies (*n* = 4). The authors sought 103 full-text articles for further consideration. Two articles could not be obtained, and 30 additional articles did not satisfy review inclusion criteria, such as not reporting on the association between an area-level variable and alcohol-related mortality, or not providing sufficient data for analysis. Thus, 71 articles were included in the detailed review (see the flow diagram^21^ in Figure 2).

## Results of the Reviewed Studies

### Alcohol-Related Mortality Outcomes

The reviewed studies most commonly assessed mortality at the county level (37 studies) or the state level (31 studies). Despite the importance of community-level SDOH,^8^,^9^ only three studies used smaller areas of aggregation such as Census tracts or zip codes. This shortcoming of the literature limits understanding of how local factors influence alcohol-related mortality. Accordingly, there is an opportunity for future research to explore associations at smaller scales, such as census tracts or neighborhoods, to inform more targeted interventions and local policy solutions.

Table 2 summarizes the different alcohol-related mortality outcomes across the levels of aggregation. The most commonly assessed outcome—by far—was alcohol-involved MVC fatalities, which were examined in 16 state-level studies^22^–^38^ and 11 county-level studies.^39^–^48^ Cirrhosis and alcohol-related liver disease mortality were the most common chronic health outcomes studied and were evaluated in two neighborhood-level studies,^49^,^50^ nine county-level studies,^51^–^59^ and two state-level studies.^60^,^61^ Relatively few studies examined mortality related to other diseases caused by chronic alcohol consumption, such as cancer,^62^ or other conditions with alcohol as a contributing cause.^63^–^67^ Several studies combined alcohol- and other drug-related mortality,^68^–^70^ at times adding suicide as a measure of deaths of despair,^71^–^76^ whereas others did not specify the exact underlying causes or included many different causes in a composite alcohol-related mortality outcome.^5^,^77^,^78^ In the sections that follow, results of reviewed studies are grouped for specific outcomes (e.g., alcohol-involved MVC fatalities) to summarize cause-specific relationships with SDOH and are compared across different outcomes to demonstrate the breadth of impacts of SDOH on causes of alcohol-related mortality.

### Area-Level Variables

Area-level variables were coded into categories of predictors based on the guiding taxonomy. The authors specifically coded indicators of political context; policy context; racial and ethnic segregation, diversity, or discrimination; socioeconomic factors; health care and social services availability and accessibility, including alcohol treatment and other mental and physical health care resources; social factors, including social capital, social connection, and social isolation; alcohol prevention activities; alcohol availability; and built environment factors, such as urbanicity and road infrastructure. Some factors may impact mortality at multiple levels (e.g., state, county, and community economic contexts), while others may be concentrated at specific levels (e.g., state alcohol policies, a community’s built environment).

The majority of studies used multiple years of mortality data, with study designs falling primarily into two categories. Cross-sectional designs using multiple years of data focused on population associations (e.g., economic disadvantage with mortality). Retrospective longitudinal designs focused on specific temporal trends for a specific unit of analysis (e.g., mortality for states that adopted a policy). Appendix 1 summarizes the main characteristics and results of the reviewed studies.

Table 3 summarizes the different contextual variables across the levels of aggregation. Most state-level studies (25 of 30) examined some dimension of alcohol or drug policy in relation to mortality,^22^,^24^–^31^,^33^–^38^,^61^–^63^,^66^,^67^,^77^,^79^–^82^ with some studies taking advantage of lengthy time series data to capture fluctuations in mortality rates in response to policy changes in specific states.^22^,^33^,^63^,^67^,^77^ Other studies capitalized on between-state variability in policies over time.^24^–^26^,^28^,^30^,^31^,^34^–^38^,^61^,^62^,^66^,^79^–^82^

Some county-level studies also focused on alcohol or drug policies,^42^,^45^,^47^,^48^,^64^,^83^,^84^ but most county-level studies (16 of 38) addressed built environment characteristics such as urbanicity or population demographic distributions.^23^,^39^–^41^,^51^,^53^,^57^,^59^,^65^,^68^–^70^,^73^,^75^,^85^,^86^ Several county-level studies examined multiple area-level characteristics.^72^,^76^,^78^ Overall, 26 studies addressed area-level socioeconomic status (SES),^26^,^37^,^40^,^44^–^47^,^49^,^50^,^54^–^56^,^58^,^60^,^68^,^71^,^72^,^74^,^76^,^77^,^86^–^90^ although only 9 studies centered SES as a focal contextual variable and the other 17 studies included SES as a covariate. As discussed below, most of these analyses were descriptive, and very few studies examined specific mechanisms of action linking the built environment or socioeconomic SDOH with the mortality outcomes. Notable results on the SDOH represented in the reviewed studies that were examined as focal contextual variables or covariates are presented in the sections that follow and in Appendix 1.

### Alcohol and Drug Policies

Thirty-two studies identified by the review focused on relationships between alcohol (and occasionally, drug) policies with mortality outcomes. In many studies, singular alcohol policies were evaluated, particularly for their association with alcohol-involved MVC fatalities. These analyses showed lower mortality rates associated with laws such as 0.08% legal blood alcohol concentration (BAC) limits for driving,^29^ Sunday sales bans in New Mexico,^45^ higher beer taxes,^82^ ignition interlock requirements for DUI offenders,^31^,^36^,^37^ minimum legal drinking age of 21 years,^24^ zero-tolerance laws (ZTLs; making it illegal for any driver under age 21 to have a BAC greater than 0.00%),^22^ greater police enforcement of DUI laws (measured by DUI arrests),^47^ and administratively revoking licenses of DUI offenders.^27^ One study using synthetic control methods found that a spirits tax increase in Illinois was associated with a temporary reduction in alcohol-involved MVC fatalities but only in counties that did not border another state.^33^ A recent study using a data set spanning from 1986 to 2005^48^ compared the impact of several state- and county-level policies on alcohol-related MVC deaths using longitudinal state-level fixed-effects models (which assess how policy changes within states impact mortality outcomes over time), difference-in-differences models (which assess how trends vary across areas with different policies), and fixed-effects models for pairs of contiguous counties located in different states (which assess policy impacts in areas that are geographically similar). The authors concluded that, at the state level, the most effective policies were beer taxes, open container prohibitions, and higher fines for DUI offenses, while at the county level, the most effective policies were ZTLs, open container prohibitions, and license revocation for DUI. These policies were significantly associated with reductions in alcohol-involved MVC deaths. Other policies, such as 0.08% BAC limits, keg registration laws, mandated community service for DUI offenses, and mandatory jail sentences for DUI offenses, were not significantly associated with either state- or county-level MVC deaths in models that also included the aforementioned effective policies.

Evidence suggests that alcohol policies also impact alcohol-related mortality due to causes other than MVC. One investigation^79^ showed that prohibition laws from 1900 to 1920 were associated with significant reductions in alcohol-related mortality attributed to diseases (e.g., circulatory disease, cirrhosis, liver disease) and to other causes (e.g., accidents, homicides, suicides). Evaluation of South Dakota’s 24/7 Sobriety program for repeat DUI offenders^91^ showed a reduction in both all-cause mortality and deaths attributed to circulatory diseases likely to be alcohol-related.^64^ One national study^82^ found that increased wine taxes reduced alcohol-involved suicide and alcohol-attributed deaths due to falls. Further, tax increases on alcohol beverages reduced mortality from alcohol-related diseases (excluding injuries) in New York,^77^ Alaska,^67^ and Florida.^63^ By contrast, another national study^66^ found that, rather than beer or spirits tax rates, government control of spirits sales was associated with reduced mortality from alcohol-related diseases. Finally, increased density of both on-premise and off-premise alcohol outlets was associated with alcohol-involved suicides in 14 U.S. states, and these effects were particularly strong for men and American Indian and Alaska Native decedents.^83^

Conflicting evidence regarding policy impacts also exists, however. Using difference-in-differences models, Freeman^27^ showed a lack of evidence of effectiveness for 0.08% BAC limits on weekend nighttime MVC fatalities (presumed to be alcohol-related), although that study did not have data on actual alcohol-involved MVC incidents. Studies also failed to detect relationships between a county’s status as “dry” (no alcohol sales), “moist” (some local restrictions on sales), or “wet” (alcohol widely available) and alcohol-involved MVC fatalities nationally,^46^ or with alcohol-related homicide victimization in Kentucky.^84^ Weak evidence was found for impacts of beer taxes on MVC fatalities for young adults ages 18 to 20.^24^ Finally, higher spirits taxes were associated with increased alcohol-involved deaths from falls in a different study,^82^ which those authors attributed to substitution effects (i.e., as spirits prices increase, people may purchase other alcohol beverages).

Some other studies revealed complex associations of alcohol policies with mortality. For example, one study^30^ documented interactive effects of DUI arrests with both 0.08% BAC limits and ZTLs on alcohol-related MVC fatalities, suggesting that stronger alcohol control policies must be actively enforced to be effective. In another study, a California state law banning sales of both alcohol and gasoline at a given site (e.g., a gas station) was associated with reduced alcohol-related MVC fatalities.^42^ However, at the same time, analyses of this policy’s effects in five counties in the Los Angeles area also suggested that some locations in suburban areas experienced an increase in alcohol-related MVCs causing property damage, and some locations in urban areas experienced an increase in serious injury.^42^ Thus, some policies may have unintended consequences impacting outcomes other than mortality.

A few studies focused on how other drug policies contributed to alcohol-involved mortality. For example, a study using data from 1982 to 1988^81^ found that increased cigarette taxes were associated with reduced mortality where alcohol was a contributing cause of death (including oral and liver cancers), but not with deaths where alcohol was the primary cause (e.g., alcohol-related cirrhosis), suggesting some specificity in the effects. A recent study^68^ found states that had laws ensuring access to the overdose reversal drug naloxone had a reduced relative risk of deaths attributable to alcohol-involved polysubstance use (opioids plus alcohol and benzodiazepines) compared to those attributable to opioids alone. Another study examined deaths of despair in the state of Illinois,^72^ documenting that alcohol-related deaths were positively associated with the opioid prescribing rate and with drug arrest rates. These findings suggest that policies targeting substances other than alcohol may help reduce alcohol-related mortality.

Five studies considered the joint effects of multiple policies simultaneously. Fixed effects regression models using data from 1982 to 1988 showed increased alcohol prices were associated with reductions in deaths where alcohol is a contributing cause, but not with deaths where alcohol is a primary cause.^81^ Those models also accounted for mandatory jail sentences for DUI and dram shop liability laws (i.e., laws that hold businesses liable for harm caused by individuals who were served or sold alcohol at the establishment), neither of which were associated with mortality due to alcohol as either a primary or contributing cause. Another study^38^ found that three key alcohol safety laws—license revocation for DUI, 0.10% BAC, and 0.08% BAC—jointly were associated with a significant downward trend in fatal alcohol-involved MVCs between 1982 and 1997. Two studies by Fell and colleagues examined 16 laws targeting underage alcohol use and DUI. One study found that possession and purchase laws and the strength of false identification laws were associated with reductions in alcohol-involved MVC fatalities among drivers under age 21.^25^ The other study^26^ showed that a suite of four laws targeting underage alcohol use and DUI (possession, purchasing, use and lose [suspended driver’s license with an underage drinking violation], and ZTLs) as well as three laws targeting all drivers (0.08% BAC, license revocation, and primary seat belt laws) were associated with significant declines in alcohol-involved MVC fatalities among drivers under age 21. Additionally, the latter set of laws was associated with reductions in these fatalities among drivers age 26 or older as well. Scherer and colleagues^35^ also found that both dram shop liability and responsible beverage service training were associated with reductions in alcohol-involved MVC fatalities in drivers under age 21, even when accounting for minimum legal drinking age laws and other DUI-related policies (including 0.08% BAC).

In a series of studies, researchers used the composite alcohol policy scores (APS) developed by Naimi and colleagues^92^ to describe the strength of a state’s combined alcohol policy environment in relation to different alcohol-involved mortality outcomes. Stronger alcohol policy environments (indicated by higher APS) were associated with reduced rates of alcohol-related cirrhosis deaths nationally among women (but not among men) and among all racial and ethnic groups other than American Indian and Alaska Native people^61^ as well as with reduced rates of alcohol-attributable cancers.^62^ These environments also were associated nationally with reduced alcohol-involved MVC fatalities among males and females under age 21, including deaths of drivers and passengers,^28^ and with reduced alcohol-involved MVC fatalities among people age 21 or older, including crashes involving driver(s) with BAC greater than 0.00% but less than 0.08%.^34^ Finally, higher APS were associated with reduced alcohol-related homicide victimization among people in 17 states, including reductions in firearm homicides and those related to intimate partner violence.^80^ Of note, one study^93^ documented a trend of nationally increasing APS from 1999 to 2018, which the authors attributed to increased stringency of laws pertaining to alcohol-impaired driving.

Overall, evidence supports the beneficial effects of alcohol policies such as higher alcohol taxes, ZTLs, license revocation, and fines for DUI on alcohol-related mortality. These effects include both reductions in alcohol-involved MVC fatalities and deaths attributed to alcohol-related diseases (including but not limited to cirrhosis and liver disease). Evidence is less robust for mandatory jail sentences for DUI, although enforcement of DUI restrictions (as indicated by DUI arrests^30^) appears to be important for reducing alcohol-related MVC fatalities. Future research may consider sub-state variations in policy effects as well as subgroup differences in impacts on alcohol-related mortality. Continued updates to composite measures of state-level alcohol policy strength (such as the APS^92^) would enable comparison of future research with the large extant evidence base that encompasses diverse causes of alcohol-related mortality.

### Socioeconomic Factors

The most common measures of socioeconomic SDOH assessed in the studies identified in this review were median household income or proportion of the population living below poverty level, with some variability in associations with the mortality outcomes. Descriptive analyses of national data showed states in the highest quartile of chronic liver disease mortality had a lower median income compared with states in the lower mortality risk quartiles.^60^ A study of New York State (excluding New York City) found that alcohol-related disease mortality was inversely associated with state-level per capita personal income over time.^77^ At the zip code level, one study of New York City found that a 10% increase in area-level poverty was associated with a 10% increase in alcohol-poisoning deaths,^89^ another study of New York City neighborhoods found higher poverty rates were associated with increased liver disease mortality,^49^ and data from both New York and California counties showed cirrhosis mortality was associated with lower SES.^54^ Moreover, another study found that higher county-level poverty was associated with a higher likelihood of opioid-related suicides involving alcohol compared with opioid-only suicides.^68^ However, several other studies did not detect such associations. A study of four states in the U.S.-Mexico border region found no association between county-level alcohol-related mortality and poverty rates, proportion of county residents with less than a high school education, or unemployment rates.^86^ Two studies examining the association between economic factors and alcohol-involved MVC fatalities found that lower county-level poverty^47^ and higher income per capita^37^ were associated with a higher number of alcohol-related fatal MVCs. Another study focusing on major metropolitan areas did not detect an association between median household income and alcohol-involved MVC deaths.^40^

Other indicators of socioeconomic context included unemployment and educational attainment. Unemployment was associated with more alcohol-related fatal MVCs among drivers under age 21 nationally^25^ and among drivers in New Mexico^45^ and Idaho,^44^ as well as with increases in deaths due to cirrhosis and chronic liver disease,^58^ and deaths from acute causes (e.g., alcohol poisoning and alcohol-involved suicide).^90^ Kerr and colleagues^87^ found that unemployment rates were associated with reduced alcohol-involved suicide rates for some groups (men ages 45 to 64 and women age 65 or older), but they also noted that these associations were no longer significant when adjusting for poverty rates. Five studies also reported associations between educational measures and alcohol-related mortality, with three studies finding that the proportion of residents with a bachelor’s degree or higher was associated with lower mortality^44^,^47^,^76^ and two studies finding no statistical significance.^40^,^86^

A few studies looked at economic security or SES as a composite measure. Knapp and colleagues^88^ found that deaths due to alcohol poisoning and chronic liver disease from 2000 to 2015 were higher in counties with greater economic insecurity, and Khatana and Goldberg^55^ showed increases in economic prosperity were associated with reductions in chronic liver disease mortality. Another study of six states and two metropolitan areas found that neighborhood socioeconomic deprivation was associated with increased risk of chronic liver disease mortality.^50^ One national study found that a stronger economy was associated with a lower ratio of alcohol-involved fatal MVCs.^26^ Again, however, not all studies identified such associations. A study based in Illinois did not find an association between the composite Distressed Communities Index^94^ and overall alcohol-related mortality at the county level.^72^ Similarly, Stringer found no association between county-level alcohol-related MVC fatalities and a composite measure of SES.^46^ Finally, Pierce and Schott found no association between the impact of permanent normal trade relations (based on proportion of the county workforce affected by manufacturing and agricultural import tariffs) and county rates of alcohol-related liver disease.^56^

Only four studies looked at how economic SDOH may interact with other factors. One study found that the association between age distributions and deaths of despair was moderated by median county income in Florida,^76^ such that alcohol-related mortality was positively associated with median age only in counties with lower median income. Another study found differences in associations of county-level poverty with alcohol-involved suicides by the decedent’s age group: Higher county-level poverty was associated with a higher likelihood of alcohol involvement in suicide among men ages 45 to 64, but with a lower likelihood among men ages 20 to 44.^87^ One study found no associations between county median income or county poverty and alcohol-related mortality when all counties from 46 states were analyzed together, but did find that a higher percentage of the population in poverty was associated with more alcohol-poisoning deaths in urban counties.^71^ Finally, Seto and colleagues found that the association between religiosity and deaths of despair varied by socioeconomic deprivation.^74^ In counties with lower economic disadvantage, six of the eight measures of religiosity (adherence and congregation size per capita for four different religions) were not correlated with deaths of despair, while two measures (percentage adherence to mainline Protestantism and percentage Catholic) were negatively associated. However, in highly disadvantaged counties, the percentage adherence to mainline Protestantism and both percentage Catholic and larger Catholic congregation size per capita were positively associated with deaths of despair, whereas both percentage adherence to Black Protestant churches and larger congregation size per capita were negatively associated with deaths of despair.

Overall, research suggests poor economic conditions are associated with higher rates of alcohol-related mortality, particularly deaths due to alcohol-involved cirrhosis and/or liver diseases. However, there was substantial variability in measures used to assess economic conditions and in the units of analysis. Future research into associations between economic conditions and alcohol-related mortality would benefit from including measures comparable with prior studies (e.g., median household income, unemployment rates, percentage in poverty, proportion of residents with a college degree, and composite measures of economic security or prosperity) and contrasting effects of economic conditions at different spatial levels (state, county, and community). Few studies explicitly examined explanations for associations of socioeconomic SDOH with alcohol-related mortality outcomes—other than noted exceptions of studies by Khatana and Goldberg,^55^ who explored the role of access to health care in disadvantaged counties, and by Major and colleagues,^50^ who explored the role of both alcohol outlet density and health care access in disadvantaged communities. Conceptually driven studies focused on mechanisms of action would greatly advance knowledge of how socioeconomic SDOH impact specific causes of alcohol-related mortality.

### Built Environment Characteristics

County-level urbanicity/metropolitan status was the most common built environment characteristic included as a focal variable^23^,^39^,^53^,^57^,^65^,^68^,^70^,^73^,^75^ or covariate.^25^,^40^,^46^,^47^,^55^,^72^,^86^ The associations between urbanicity/metropolitan status and alcohol-related mortality varied substantially. Most studies found that alcohol-related mortality—including DUI fatalities,^23^,^25^,^39^,^46^ combined alcohol- and other drug-related mortality,^68^,^70^ deaths of despair,^73^ cirrhosis/liver disease mortality,^53^,^57^ and other chronic alcohol mortality^65^—was higher in rural counties. In contrast, three studies using mortality data from 2010 onward found that mortality from multiple causes^72^,^86^ and cirrhosis/liver disease mortality^55^ were higher in urban areas. Four studies found a nonsignificant relationship between urbanicity/metropolitan status and DUI fatalities^40^,^47^ and deaths of despair.^72^,^75^ The heterogeneity in these results suggests there may be important effect modifiers for further consideration.

Several studies used total/daily vehicle miles traveled (a proxy for traffic volume) as a covariate in models examining the relationship between DUI fatalities and area-level predictors.^25^,^44^,^46^,^47^ In each study, more vehicle miles traveled were associated with higher DUI fatality rates. Relatedly, the availability of ridesharing was assessed as a potential mechanism to reduce DUI fatalities. Brazil and Kirk^40^ found that overall Uber availability was not related to DUI fatalities, but it was associated with more DUI fatalities in population-dense or urban areas, perhaps due to an increase in traffic volume.

Other characteristics of the built environment also affected alcohol-mortality rates. Cotti and Walker^41^ found that casino openings were related to more DUI fatalities both in the county in which the casino was located and in neighboring counties. Zemore and colleagues^86^ found that alcohol- and other drug-related mortality was highest in off- versus on-border counties in the four U.S.-Mexico border states, despite off-border counties having higher proportions of college-educated residents and a lower likelihood of being designated as a high-intensity drug trafficking area.

Several other studies found regional variation in alcohol-related mortality outcomes;^5^,^51^,^59^,^69^ however, explanations were not tested empirically. Seto^74^ found that Appalachian status, percentage of veterans, and economic reliance on mining as employment (relative to nonspecialized) were all positively associated with deaths of despair in U.S. counties, although a county’s economic reliance on farming and manufacturing for employment was negatively associated with deaths of despair. These employment factors may help explain regional variation in alcohol-related mortality as well.

Future studies could advance interventions to improve community health by explicitly examining mechanisms contributing to urban and rural differences in mortality outcomes, as there may be specific drivers of cause-specific deaths. For example, physical and mental health care access and economic disinvestment may contribute to deaths due to chronic heavy alcohol use in rural communities, while other SDOH such as alcohol outlet densities and social connection may be more relevant in urban and suburban areas.

### Other Domains

#### Health care and social services

Some studies included measures of health care and social services, which are important determinants of mortality.^95^ Six studies reported associations between area-level health care factors and alcohol-related mortality outcomes. Two studies focused on deaths of despair,^71^,^76^ two examined alcohol-related MVC fatalities,^32^,^43^ and two examined liver-related mortality.^52^,^55^ No studies focused on social services factors per se, although one study of U.S. state preemption laws that constrain local governments’ ability to enact legislation to raise the minimum wage or mandate paid sick leave^90^ found statistically significant associations between availability of paid sick leave and reductions in fatal alcohol poisonings for women.

Bradford and Bradford,^71^ in a study investigating the relationship between county-level eviction rates and combined alcohol- and other drug-related mortality rates, used the number of active physicians per 1,000 county residents and percentage of residents without health insurance as covariates. The number of active physicians was positively associated with alcohol poisoning in models including data for all counties nationally, but this variable did not remain statistically significant when analyses were stratified by urbanicity. The percentage of the population without health insurance was not statistically significant in any of their models. Zeglin and colleagues^76^ found that above-average rates of regular medical care (e.g., proportion of adults with recent medical checkups) were associated with fewer deaths of despair, but above-average mental health care availability (e.g., number of licensed social workers, psychologists, marriage/family therapists, and counselors per 10,000 residents) was unexpectedly associated with more deaths of despair. The percentage of adults with health insurance coverage and public health department expenditures were not significantly related to county-level deaths of despair, however.

Freeborn and McManus^43^ evaluated whether the county-level number of substance use treatment clinics was associated with alcohol-related MVC fatalities in non-metropolitan counties across the United States. Predictive models estimated that each additional inpatient or residential clinic was associated with 15% fewer alcohol-related MVC fatalities, while each additional outpatient clinic was associated with 26% fewer alcohol-related MVC fatalities in the county where the additional clinic was located. Nonsignificant findings in models predicting overall MVC fatalities suggested that the effects of county-level substance use treatment availability were specific to alcohol-related fatalities. Using national data, Mann and colleagues^32^ tested whether the number of Alcoholics Anonymous (AA) members and number of people receiving any alcohol or drug treatment were associated with state-level alcohol-related MVC fatalities. Higher AA membership was associated with lower rates of alcohol-related MVC fatalities, but the number of people receiving alcohol or drug treatment was unrelated to MVC fatalities.

Goldberg and colleagues^52^ found liver disease mortality was higher in counties with a greater proportion of uninsured adults and in counties located farther away from a liver transplant center. Counties with higher transplant wait-listing rates paradoxically had lower liver disease-related mortality rates. Gastroenterologist access was not significantly associated with liver disease mortality. Finally, in their study of county-level economic prosperity and liver disease-related mortality among U.S. adults, Khatana and Goldberg^55^ found that the percentage of insured individuals and number of primary care providers were not statistically significant predictors, although a larger number of county hospital beds was associated with higher liver disease-related mortality.

Although associations between health care and social services factors and alcohol-related mortality were mixed, some patterns did emerge. Indicators of health care use^32^,^76^ were more strongly related to alcohol mortality outcomes than were indicators of general health care availability.^52^,^55^,^71^ This might be because health care service availability does not necessarily guarantee health care access or indicate that such access is equitable across individuals with varying risk for alcohol-related mortality. Another conclusion from this small set of studies is that health care factors protected against negative alcohol use consequences more strongly as they became more specific to alcohol use.^32^,^43^ This might partially explain the observation that health insurance coverage was only significantly related to alcohol mortality in one of four studies that accounted for this factor.^52^ It could be that insurance plans did not sufficiently cover prevention or treatment services for alcohol use and associated consequences. The single study that focused on health policies and alcohol-related mortality^96^ found reductions in deaths caused by acute effects of alcohol (e.g., alcohol poisonings) after the implementation of California’s Mental Health Services Act in 2006, which the authors attributed to improvements in access to prevention and treatment.

Two studies found that greater health care access was associated with worse alcohol-related mortality outcomes.^55^,^76^ One potential explanation is that services are made more available and providers choose specific geographic markets precisely because alcohol-related problems are more prevalent in that area. As most of these studies were retrospective and cross-sectional, however, inferences regarding causality or directionality are limited. Longitudinal studies testing mediation pathways could advance understanding of how health care and social services may reduce alcohol-related mortality.

#### Racism, discrimination, and racial or ethnic composition

Racism and discrimination are key SDOH and drivers of alcohol-related health inequities.^97^–^99^ To date, the strongest evidence linking racial discrimination to health inequities in the United States is through discrimination’s adverse effects on psychological wellbeing, mental health, and related health practices, including alcohol use.^100^ Yet no studies of alcohol-related mortality included formal measures of racism or discrimination, and relatively few studies included related indicators, such as an area’s racial or ethnic group composition, including percentage of White/Caucasian or non-Hispanic residents;^47^,^71^,^74^ percentage of non-Hispanic Black or African American residents;^37^,^47^,^55^,^74^,^86^ percentage of Hispanic or Latinx residents;^47^,^55^,^74^,^78^,^86^ or percentage of residents of another racial or ethnic group.^37^ Of note, racial and/or ethnic group composition was always included as a covariate and never an exposure of interest.

Findings on area-level racial and/or ethnic group composition in relation to alcohol-related mortality were quite mixed. One study found lower rates of alcohol-involved mortality in counties that had higher proportions of Hispanic residents with low levels of acculturation.^78^ Similarly, other studies found that a higher percentage of non-Hispanic Black people in an area was associated with fewer cirrhosis/liver disease^55^ and DUI fatalities.^37^ Seto^74^ found that a high relative concentration of three major racial or ethnic groups (Caucasian, African American, Hispanic) each was negatively associated with deaths of despair, but another study found no statistically significant associations of racial or ethnic composition with deaths of despair.^71^ Using data from states in the U.S.–Mexico border region, Zemore and colleagues^86^ found that higher county-level percentages of Black and Latinx people were associated with less drug mortality and less combined alcohol- and other drug-related mortality, but not with alcohol-related mortality when considered alone, suggesting there may be different determinants of drug-related and alcohol-related deaths. By contrast, another study found that the proportion of Hispanic residents was associated with higher risk of unspecified alcohol mortality.^78^ Finally, Stringer^47^ found that county proportions of residents who were Caucasian, African American, or Hispanic were not significantly related to DUI fatalities when assessed with linear models, although each of these racial or ethnic composition variables was associated with lower mortality risk in quadratic models, suggesting that race and ethnicity may have complex relationships with alcohol-related mortality. Of note, most of these studies compared mortality outcomes of areas with high proportions of certain racial or ethnic group residents (e.g., Caucasian, African American, Hispanic) with those in areas with high proportions of other populations who do not identify as any of the listed groups (e.g., American Indian and Alaska Native people), rather than directly testing associations of American Indian and Alaska Native resident density with mortality outcomes. However, one early study using data from the 1980s noted that alcohol-related mortality was higher among urban American Indian and Alaska Native people than among White people in Washington state.^85^

Studies that explicitly operationalize area-based measures of structural racism, including segregation and redlining, could help the field move beyond purely descriptive analysis of racial and ethnic composition in relation to alcohol-related mortality. Analysis of the political context also was lacking, and studies of how gerrymandering and state actions designed to increase or decrease racial and ethnic segregation may impact mortality due to alcohol use also would be informative. In future research, attention to both subgroup differences and pathways from racism and discrimination to alcohol-related mortality would advance efforts to improve community health.

#### Social norms and social control of high-risk alcohol use

Area-level drinking cultures may either increase or decrease alcohol-related mortality risks. For example, higher proportions of young residents (particularly young men) in an area might foster social norms encouraging heavy alcohol consumption, whereas higher proportions of certain religious groups might discourage alcohol consumption. Ransome and colleagues^89^ found that an increase in area-level prevalence of heavy drinking in New York City was associated with higher subsequent risk of alcohol-poisoning deaths. Similarly, one national study showed that county-level per-capita alcohol consumption was a significant predictor of DUI fatalities.^47^ However, in another study conducted in the 100 largest metropolitan areas in the United States, a county’s percentage of adults reporting recent binge drinking (defined as five or more drinks per occasion for men and four or more drinks for women) or heavy alcohol use (defined as 15 or more drinks per week for men and eight or more drinks per week for women) was not significantly associated with DUI fatalities.^40^ Stringer^46^ found that increases in anti-alcohol community norms, values, attitudes, and beliefs were related to decreases in alcohol-related MVC fatalities, and Ahern and colleagues^49^ showed that higher levels of social control were associated with lower rates of liver disease mortality in New York City neighborhoods.

Several studies assessed the relationships of mortality outcomes with area-level demographic correlates, yielding mixed results. Other than the findings related to socioeconomic factors and racial and ethnic composition reviewed in the previous sections, results for aggregated demographic characteristics are not reviewed in depth, as these measures are difficult to interpret in relation to SDOH. Appendix 1 indicates studies that considered an area’s age distribution^40^,^47^,^71^,^72^,^74^ or gender or sex distributions^37^,^44^,^47^,^53^,^55^,^71^,^72^,^74^ in relation to the mortality outcomes. Theoretically driven future research could provide more meaningful investigation of how demographic composition might either cause or reduce alcohol-related mortality, such as through social norms related to alcohol use or attitudes about driving after drinking. Multilevel analyses may be most informative for addressing these questions.

## Discussion

This review synthesizes research on area-level SDOH associated with alcohol-related mortality. Although some of these determinants are shared with drug overdose deaths (such as socioeconomic disadvantage), others are more specific to mortality due to acute and/or chronic alcohol use (such as alcohol control policies). Research published since 1990 has studied a wide variety of alcohol-related mortality outcomes at different levels of analysis, using diverse analytic strategies and varied sets of covariates, and using different years of data from various places across the country.

Several limitations should be noted regarding how area-level factors were assessed across studies. For example, findings summarized here were limited to area-level relationships (ecological analyses) and did not include multilevel analyses assessing impacts of contextual determinants on individual-level mortality risk. Additionally, with the exception of most of the alcohol policy studies, many studies were cross-sectional or descriptive, limiting causal inferences for the effects of many SDOH. There also was wide variability in the degree to which specific mortality causes were examined in relation to SDOH. For example, although many studies analyzed alcohol-related MVC or cirrhosis/liver disease fatalities, fewer studies analyzed other mortality causes due to chronic alcohol consumption. Additionally, many studies relied on derived measures^101^ based on aggregated characteristics of individuals or households in an area (e.g., county-level median income, proportion of people without a college degree, proportion of residents from a specific racial or ethnic group) rather than using integral measures^101^ of the area’s structural characteristics (e.g., descriptions of the health care or education environment, measures of racism or redlining). Although both types of measures can provide valuable information on geographic differences in mortality, the latter provides more direct assessment of associations with fundamental SDOH.^10^ Finally, only seven articles^44^,^45^,^48^,^51^,^74^,^86^,^89^ incorporated statistical methods to address spatial autocorrelation—that is, the associations between adjacent or nearby spatial units of analysis (e.g., county, Census tract). Ignoring spatial autocorrelation may lead to incorrect statistical inferences because the assumption of independence is violated.^102^ Future mortality studies could assess spatial autocorrelation and address it analytically if needed.

Overall, this review found that the literature in most of the thematic areas addressed is theoretically underdeveloped. Consistent with the primary conclusion of a smaller review of social determinants of deaths of despair,^19^ future research could focus on a more diverse set of SDOH and area-level predictors of alcohol-related mortality in community health research and prevention. As few studies examined major health policy changes or focused on health care system factors beyond inclusion as covariates, prospective studies could better disentangle effects of care availability and access on alcohol-related mortality outcomes by examining factors such as the prevalence of different treatment models (e.g., abstinence, harm reduction, integrated physical and behavioral health care) and approaches (e.g., pharmacotherapy, cognitive behavior therapy, community support). Further, few U.S. states have restrictive alcohol policy environments;^93^ therefore, future work examining sub-state variation in alcohol policies and impacts on alcohol-related mortality may yield useful findings. Policies targeting other substances also may contribute to reduced alcohol-related mortality, and these policies may interact with health care services as well.

This review did not identify any studies that advanced the understanding of how racism and discrimination, community-level prevention activities, or social services relate to alcohol-related mortality. Further work could help to better characterize the specific social determinants of increased alcohol-related mortality in Indigenous communities, such as increased alcohol availability or targeted marketing tactics.^103^ Recent research examining the impact of state-level structural racism on alcohol use behaviors found that some dimensions of structural racism (e.g., incarceration segregation) but not others (e.g., residential segregation, economic segregation) were related to increased alcohol use.^104^ Moreover, discrimination, often measured as an interpersonal psychosocial stressor, is associated with increased alcohol consumption.^99^ Future studies could directly assess relationships of structural racism and discrimination with alcohol-related mortality attributed to different causes (both acute and chronic) while considering new and alternative measures of racism and discrimination at different geographic scales.

Additional studies could provide insights into the link between local community and neighborhood contexts and alcohol-related mortality, given that preventive interventions are more likely to be implemented at the local level than are policy changes (commonly addressed by states) or health care system improvements (typically addressed by states and counties). Future work also could explore subgroup differences, interactions between different SDOH, and specific mechanisms of action to identify strategies to improve population health. Given the length of time it takes to see the effects of SDOH interventions on mortality, simulation models may allow cost-effective exploration of potential benefits of combinations of interventions, as well as variation in impacts across geographic contexts and for high-priority demographic subgroups.

**KEY TAKEAWAYS**

- The literature on social determinants of alcohol-related mortality includes many studies focused on area-level determinants of alcohol-involved motor vehicle crashes and cirrhosis or liver disease mortality.
- Extant research highlights the benefits of stronger alcohol policies and the importance of socioeconomic conditions as determinants of alcohol-related mortality.
- Substantial gaps in knowledge remain, particularly related to potential impacts of structural racism, community-level prevention, and community social and medical services on alcohol-related mortality.

## Figures and Tables

**Figure 1:**
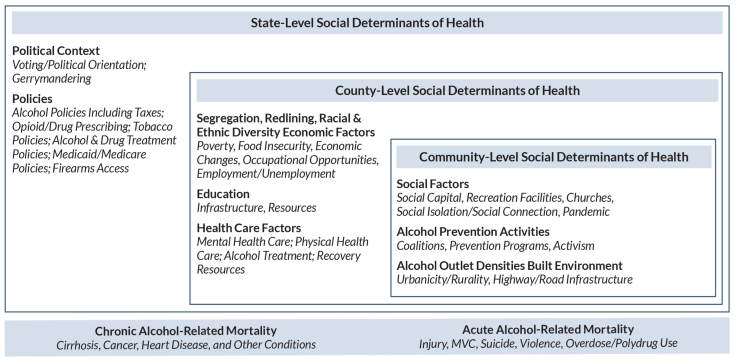
Social determinants of chronic and acute alcohol-related mortality at various levels of aggregation.

**Table 1:** Search Strategy Details, by Database

PubMed
#1 ((“census tract*”[tiab] OR “zip code*”[tiab] OR “ZCTA”[tiab] OR “ZCTAs”[tiab] OR neighborhood*[tiab] OR “county”[tiab] OR “counties”[tiab] OR “state-level”[tiab] OR “state”[ti] OR “area-level”[tiab] OR “Census Tract”[MeSH]) AND (alcohol*[ti] OR “alcohol-related”[tiab] OR “alcohol-involved”[tiab] OR “alcoholic hepatitis”[tiab] OR “cirrhosis”[tiab] OR ((“liver disease*”[tiab] OR “fatty liver”[tiab]) AND alcohol*[tiab]) OR (“deaths of despair”[tiab] AND (“mortality”[tiab] OR “liver disease*”[tiab] OR “cirrhosis”[tiab]))) AND (“mortality”[tiab] OR death*[tiab] OR fatal*[tiab] OR poisoning*[tiab] OR decedent*[tiab] OR “died”[tiab] OR suicide*[tiab] OR “Alcohol-Related Disorders/mortality”[MeSH]) AND (“United States”[MeSH] OR “United States”[Title/Abstract] OR “USA”[Title/Abstract] OR “U.S.A.”[Title/Abstract] OR “U.S.”[Title/Abstract] OR “United States”[Affiliation] OR “USA”[Affiliation] OR “U.S.A.”[Affiliation] OR “U.S.”[Affiliation] OR “US”[Affiliation] OR “Black or African American”[MeSH] OR “Asian American Native Hawaiian and Pacific Islander”[MeSH] OR “Hispanic or Latino”[MeSH] OR “Mexican Americans”[MeSH] OR “American Indian or Alaska Native”[MeSH] OR “Indians, North American”[MeSH:NoExp] OR review*[ti] OR “Review”[Publication Type] OR “Review Literature as Topic”[MeSH] OR “Systematic Review”[Publication Type] OR “Systematic Reviews as Topic”[MeSH]) AND (“1990/01/01”[Date - Publication]: “3000”[Date - Publication])) NOT (“non-alcoholic”[tw] OR “nonalcoholic”[tw] OR (“Animals”[MeSH] NOT “Humans”[MeSH]) OR “Comment”[Publication Type] OR “Letter”[Publication Type] OR “Editorial”[Publication Type]) Filters: English
Final Result: 258 articles
Web of Science
#1 (TI=(“census tract*” OR “zip code*” OR “ZCTA” OR “ZCTAs” OR neighborhood* OR “county” OR “counties” OR “state-level” OR “state” OR “area-level”) OR AB=(“census tract*” OR “zip code*” OR “ZCTA” OR “ZCTAs” OR neighborhood* OR “county” OR “counties” OR “state-level” OR “area-level”)) AND (TI=(alcohol* OR “alcohol-related” OR “alcohol-involved” OR “alcoholic hepatitis” OR “cirrhosis” OR ((“liver disease*” OR “fatty liver”) AND alcohol*) OR (“deaths of despair” AND (“mortality” OR “liver disease*” OR “cirrhosis”))) OR AB=(“alcohol-related” OR “alcohol-involved” OR “alcoholic hepatitis” OR “cirrhosis” OR ((“liver disease*” OR “fatty liver”) AND alcohol*) OR (“deaths of despair” AND (“mortality” OR “liver disease*” OR “cirrhosis”)))) AND (TI=(“mortality” OR death* OR fatal* OR poisoning* OR decedent* OR “died” OR suicide*) OR AB=(“mortality” OR death* OR fatal* OR poisoning* OR decedent* OR “died” OR suicide*)) Timespan: 1990-01-01 to 2023-12-31 (Publication Date)
Initial Result: 394 articles
#2 #1 NOT TS=(“non-alcoholic” OR “nonalcoholic”) and English (Languages) and USA (Countries/Regions) and Review Article or Article (Document Types) Timespan: 1990-01-01 to 2023-12-31 (Publication Date)
Final Result: 234 articles
CINAHL
S1 (ti (“census tract*” or “zip code*” or “zcta” or “zctas” or neighborhood* or “county” or “counties” or “state-level” or “state” or “area-level”) or ab (“census tract*” or “zip code*” or “zcta” or “zctas” or neighborhood* or “county” or “counties” or “state-level” or “area-level”)) and (ti (alcohol* or “alcohol-related” or “alcohol-involved” or “alcoholic hepatitis” or “cirrhosis” or ((“liver disease*” or “fatty liver”) and alcohol*) or (“deaths of despair” and (“mortality” or “liver disease*” or “cirrhosis”))) or ab (“alcohol-related” or “alcohol-involved” or “alcoholic hepatitis” or “cirrhosis” or ((“liver disease*” or “fatty liver”) and alcohol*) or (“deaths of despair” and (“mortality” or “liver disease*” or “cirrhosis”)))) and (ti (“mortality” or death* or fatal* or poisoning* or decedent* or “died” or suicide*) or ab (“mortality” or death* or fatal* or poisoning* or decedent* or “died” or suicide*) or mh “alcohol-related disorders+/mo”) limiters - published date: 19900101-20231231; English Language; Peer Reviewed; exclude MEDLINE records
Initial Result: 55 articles
S2 S1 NOT (“non-alcoholic” OR “nonalcoholic” OR ZT “commentary” OR ZT “editorial” OR ZT “letter” OR ZT “letter to the editor”) Limiters - Published Date: 19900101-20231231; English Language; Peer Reviewed; Exclude MEDLINE records
Refined Result: 48 articles
S3 S2 AND (ZZ “usa” OR MH “United States+” OR ZS “usa” OR MH “African Americans” OR MH “Hispanic Americans+” OR “United States” OR “USA” OR “U.S.A.”) Limiters - Published Date: 19900101-20231231; English Language; Peer Reviewed; Exclude MEDLINE records
Final Result: 38 articles

*Note:* CINAHL, Cumulative Index to Nursing and Allied Health Literature; S1, search 1; S2, search 2; S3, search 3.

**Table 2:** Alcohol-Related Mortality Outcomes Assessed by Level of Aggregation (*N* = 71 Studies)

Specific alcohol-related mortality outcomes	Count of studies
**State-level studies (** ***n*** ** = 30)**	
Alcohol-involved MVC fatalities	16
Cirrhosis/liver disease	2
Other chronic cause	4
Alcohol-related suicide/violence	1
Other acute cause	2
Unspecified alcohol-related mortality	2
Multiple causes of death	3
**County-level studies (** ***n*** ** = 38)**	
Alcohol-involved MVC fatalities	11
Cirrhosis/liver disease	9
Other chronic cause	2
Alcohol-related suicide/violence	3
Deaths of despair	6
Alcohol- and other drug-related mortality combined	3
Unspecified alcohol-related mortality	1
Multiple causes of death	3
**Neighborhood-level studies (** ***n*** ** = 3)**	
Cirrhosis/liver disease	2
Other acute cause	1

*Note:* MVC, motor vehicle crash.

**Table 3:** Primary Area-Level Predictors Assessed by Level of Aggregation (*N* = 71 Studies)

Primary area-level predictors	Count of studies
**State-level studies (** ***n*** ** = 30)**	
Alcohol and drug policies	25
Economic context	1
Employment/work environment	1
Health policies	1
Health care and social services	1
Built environment	1
**County-level studies (** ***n*** ** = 38)**	
Alcohol and drug policies	7
Economic context	5
Employment/work environment	3
Health care and social services	2
Social context	2
Built environment	16
Multiple domains	3
**Neighborhood-level studies (** ***n*** ** = 3)**	
Economic context	3

**Appendix 1:** Characteristics of the Reviewed Studies and Selected Results

Study Authors & Year of Publication	Setting Level of Aggregation	Years Measured Outcomes Measured	Primary Area-Level Predictors	Study Design/Analysis Method(s)	Results for Focal Predictor(s) & Notable Covariates Discussed in Review
Ahern et al. (2008)^49^	New York City Neighborhood	2000 Cirrhosis/liver disease	Economic context	Cross-sectional Poisson and negative binomial regression	Key predictor: Economic disadvantage was a risk factor Covariates: Social context was a protective factor
Alattas et al. (2020)^62^	United States State	2006–2010 Other chronic cause	Alcohol & drug policies	Retrospective longitudinal/lagged Linear regression with 1-year lag from alcohol policy score	Key predictor: Alcohol & drug policies were a protective factor
Baeseman (2009)^39^	Wisconsin County	1999–2006 (pooled) DUI fatalities	Built environment	Cross-sectional (pooled data) Breslow-Day test for homogeneity of trends across urban and rural counties	Key predictor: Built environment was a risk factor
Bensley et al. (2020)^68^	27 U.S. states County	2012–2015 (pooled) AOD combined	Built environment	Cross-sectional (pooled data) Multinomial logistic regression	Key predictor: Built environment was a risk factor Covariates: Economic disadvantage was a risk factor and alcohol & drug policies had mixed effects
Blackman et al. (2001)^22^	Washington State	1990–1998 DUI fatalities	Alcohol & drug policies	Interrupted time series ARIMA with partial-trend indicator	Key predictor: Alcohol & drug policies were a protective factor
Borgialli et al. (2000)^23^	Michigan County	1994–1996 DUI fatalities	Built environment	Retrospective longitudinal Logistic regression, mediation analysis	Key predictor: Built environment was a risk factor
Bradford & Bradford (2020)^71^	46 U.S. states and District of Columbia County	2004–2016 Deaths of despair	Economic context	Observational panel analysis Linear regression with time and county fixed effects and control function (2SRI) to address endogeneity	Key predictor: Economic disadvantage had mixed effects Covariates: Health care/social services had mixed effects
Brazil & Kirk (2020)^40^	United States County	2009–2017 DUI fatalities	Built environment	Observational panel analysis Poisson regression with country fixed effects, month-by-year fixed effects, county-specific linear time trend; standard errors adjusted for county-level clustering	Key predictor: Built environment was a predictor with mixed effect
Cataldo (2022)^72^	Illinois County	2010–2014; 2015–2019 Deaths of despair	Multiple domains	Repeated cross-section Linear regression	Key predictors: Alcohol & drug policies were a risk factor and built environment had mixed effects
Cotti & Walker (2010)^41^	United States County	1990–2000 DUI fatalities	Built environment	Retrospective longitudinal/lagged Fixed effects logistic regression with robust standard errors to account for clustering of counties in states; 1-year, 2-year, and 3-year lags from casino opening	Key predictor: Built environment had mixed effect
Dee (1999)^24^	United States State	1977–1992 DUI fatalities	Alcohol & drug policies	Observational panel analysis Weighted least squares	Key predictor: Alcohol & drug policies had mixed effects
Delcher et al. (2012)^77^	New York (excl. New York City) State	1969–2006 Unspecified alcohol-related mortality	Alcohol & drug policies	Quasi-experimental time series Seasonal ARIMA; generalized linear mixed model to assess effect of tax increase or decrease on outcome	Key predictor: Alcohol & drug policies were a protective factor Covariates: Economic disadvantage additionally was a risk factor
Desai et al. (2018)^60^	United States State	2010 Cirrhosis/liver disease	Economic context	Cross-sectional Chi-square tests, Kruskal-Wallis test	Key predictor: Economic disadvantage was a risk factor
Dwyer-Lindgren et al. (2016)^51^	United States County	1980–2014 (pooled) Cirrhosis/liver disease	Built environment	Cross sectional (pooled data) Small area estimation methods, Bayesian spatially explicit mixed-effects regression model	Key predictor: Built environment was a risk factor
Farmer et al. (2005)^42^	California County	1982–1989 DUI fatalities	Alcohol & drug policies	Retrospective longitudinal Seemingly unrelated regression	Key predictor: Alcohol & drug policies were a protective factor
Fell et al. (2008)^25^	United States State	1982–1990 (pooled) DUI fatalities	Alcohol & drug policies	Cross-sectional (pooled data), between-state design ANOVA & stepwise linear regression	Key predictor: Alcohol & drug policies were a protective factor Covariates: Unemployment/work environment and built environment was risk factors
Fell et al. (2009)^26^	United States State	1982–2004 DUI fatalities	Alcohol & drug policies	Retrospective longitudinal, pre-test/post-test Structural equation modeling	Key predictor: Alcohol & drug policies were a protective factor Covariates: Economic disadvantage additionally was a risk factor
Freeborn & McManus (2010)^43^	United States County	1998, 2000, 2002–2004 DUI fatalities	Health care & social services	Repeated cross-section Linear regression with state fixed effects and standard errors adjusted for state-level clustering	Key predictor: Health care/social services were a protective factor
Freeman (2007)^27^	United States State	1980–2004 DUI fatalities	Alcohol & drug policies	Time series Two-way fixed effects specification of the pooled time series cross-section regression (DID estimators)	Key predictor: Alcohol & drug policies had mixed effects
Giesbrecht et al. (2015)^83^	14 of 17 U.S. states participating in NVDRS County	2003–2011 Alcohol-related suicide/violence	Alcohol & drug policies	Cross-sectional Correlation, multilevel logistic regression	Key predictor: Alcohol & drug policies were a risk factor
Goldberg et al. (2021)^52^	United States County	2009–2019 Cirrhosis/liver disease	Health care & social services	Cross-sectional Multivariable regression models, cluster analysis with optimized hotspot analysis	Key predictor: Health care/social services had mixed effects Covariates: Built environment was a risk factor
Grossman et al. (1994)^85^	Washington County	1981–1990 Multiple	Built environment	Cross-sectional Descriptive analyses comparing mortality rates and confidence intervals across race/urban groups	Key predictor: Built environment was a risk factor
Ha et al. (2022)^53^	United States County	1999–2019 Cirrhosis/liver disease	Built environment	Retrospective longitudinal Piecewise linear regression (Joinpoint regression) for trends	Key predictor: Built environment was a predictor with mixed effects
Hadland et al. (2015)^61^	United States State	2010–2011 Cirrhosis/liver disease	Alcohol & drug policies	Retrospective longitudinal/lagged Poisson regression accounting for state-level clustering and a 3-year lag between policies and alcohol-related cirrhosis mortality	Key predictor: Alcohol & drug policies had mixed effects
Hadland et al. (2017)^28^	United States State	2000–2013 DUI fatalities	Alcohol & drug policies	Retrospective longitudinal/lagged Logistic regression with a 1-year lag from alcohol policy score	Key predictor: Alcohol & drug policies were a protective factor
Hingson et al. (1996)^29^	Utah, Oregon, Maine, California, & Vermont compared to Idaho, Washington, Massachusetts, Texas & New Hampshire State	1976–1993 DUI fatalities	Alcohol & drug policies	Retrospective longitudinal Ratio of relative risk of alcohol-involved fatal crashes between comparison states (with 95% Cis), pre and post passage of 0.08% BAC limit in 5 U.S. states	Key predictor: Alcohol & drug policies were a protective factor
Hosseinichimeh et al. (2022)^30^	United States State	1985–2019 DUI fatalities	Alcohol & drug policies	Retrospective longitudinal/lagged Fixed-effects linear regression models with 1-year lag for DUI arrests per 100 and DUI laws	Key predictor: Alcohol & drug policies had mixed effects
Karpati et al. (2002)^54^	New York & California County	1997 Cirrhosis/liver disease	Economic context	Cross-sectional Comparison of relative mortality rates between counties in lowest and highest socioeconomic quartiles (rate ratios), Pearson’s correlations	Key predictor: Economic disadvantage was a risk factor
Kaufman & Wiebe (2016)^31^	United States State	1999–2013 DUI fatalities	Alcohol & drug policies	Retrospective longitudinal/lagged DID analysis, state fixed effects models with 3-year lag for policy adoption	Key predictor: Alcohol & drug policies were a protective factor
Kerr et al. (2017)^87^	16 U.S. states participating in NVDRS (excluding Ohio) County	2005–2011 (pooled) Alcohol-related suicide/violence	Economic context	Cross-sectional (pooled data) Generalized estimation equations (normal/Gaussian distribution, link identify function, unstructured correlation matrix)	Key predictor: Economic disadvantage had mixed effects Covariates: Unemployment/work environment had mixed effects
Khaleel et al. (2019)^84^	Kentucky County	2005–2012 Alcohol-related suicide/violence	Alcohol & drug policies	Cross-sectional Multilevel logistic regression model with random county intercept	Key predictor: Alcohol & drug policies had nonsignificant effects
Khatana & Goldberg (2022)^55^	United States County	2010–2017 Cirrhosis/liver disease	Economic context	Retrospective longitudinal Negative binomial generalized linear mixed model with random state intercept	Key predictor: Economic disadvantage was a risk factor Covariates: Health care/social services, racism/discrimination, and built environment/other spatial characteristics all had mixed effects
Knapp et al. (2019)^88^	7 U.S. states in the Mid-Atlantic region County	2001–2005; 2006–2010; 2011–2015 Multiple	Economic context	Retrospective longitudinal Log-linear mixed effects regression models with robust standard errors	Key predictor: Economic disadvantage was a risk factor
Landen et al. (2014)^69^	United States County	1999–2009 AOD combined	Built environment	Retrospective longitudinal Standardized rate ratios for American Indian/Alaska Native populations using White age-adjusted mortality rates for comparison	Key predictor: Built environment was a risk factor
Law & Marks (2020)^79^	United States State	1900–1920 Multiple	Alcohol & drug policies	Pre-test/post-test DID regression	Key predictor: Alcohol & drug policies were a protective factor
Li et al. (2019)^44^	Idaho County	2010–2015 DUI fatalities	Employment/work environment	Observational panel analysis Hierarchical Bayesian random parameters models with spatiotemporal interactions	Key predictor: Unemployment/work environment was a risk factor Covariates: Education was a protective factor, and built environment was a risk factor
Major et al. (2014)^50^	6 U.S. states (California, Florida, Louisiana, New Jersey, North Carolina, Pennsylvania) & 2 metropolitan areas (Atlanta, Georgia; Detroit, Michigan) Neighborhood	1995–2008 Cirrhosis/liver disease	Economic context	Retrospective longitudinal Multilevel/hierarchical Cox proportional hazards regression models with census tract random effects	Key predictor: Economic disadvantage was a risk factor
Maldonado-Molina & Wagenaar (2010)^63^	Florida State	1969–2004 Other chronic cause	Alcohol & drug policies	Lagged time series analysis Time series analyses including Box-Jenkins method to fit ARIMA models, fixed-effects and random-effects models	Key predictor: Alcohol & drug policies were a protective factor
Mann et al. (1996)^32^	United States State	1982 and 1990 DUI fatalities	Health care & social services	Repeated cross-section Ordinary least squares regression models using data from two time points	Key predictor: Health care/social services had mixed effects
McClelland & Iselin (2019)^33^	Illinois State	1992–2015 DUI fatalities	Alcohol & drug policies	Retrospective longitudinal Synthetic control methodology	Key predictor: Alcohol & drug policies had mixed effects
McMillan et al. (2007)^45^	New Mexico County	1990–2000 DUI fatalities	Alcohol & drug policies	Retrospective longitudinal Bayesian hierarchical binomial regression models with county random effects and adjustment for spatial pattern motor vehicle crash rates	Key predictor: Alcohol & drug policies were a protective factor Covariates: Unemployment/work environment and social context were risk factors
Mejia de Grubb et al. (2016)^78^	United States County	1999–2014 Unspecified alcohol-related mortality	Multiple domains	Repeated cross-section Joinpoint regression models for trends, ecological correlations with county demographics	Key predictors: Racism/discrimination was a risk factor and built environment was a protective factor
Monnat (2020)^73^	United States County	1990–2018 Deaths of despair	Built environment	Retrospective longitudinal Descriptive tabulations of trends for areas defined by urbanicity and by 1989 economic dependency	Key predictor: Built environment was a risk factor
Naimi et al. (2017)^80^	United States State	2003–2012 Alcohol-related suicide/violence	Alcohol & drug policies	Repeated cross-section Generalized estimating equations logistic models	Key predictor: Alcohol & drug policies were a protective factor
Naimi et al. (2018)^34^	United States State	2000–2015 DUI fatalities	Alcohol & drug policies	Retrospective longitudinal/lagged Logit generalized estimating equations with 1-year lag from policy, mediation analysis	Key predictor: Alcohol & drug policies were a protective factor
Nicosia et al. (2016)^64^	South Dakota County	2000–2011 Other chronic cause	Alcohol & drug policies	Retrospective longitudinal DID, Poisson regression	Key predictor: Alcohol & drug policies were a protective factor
Pierce & Schott (2020)^56^	United States County	1990–2013 Cirrhosis/liver disease	Employment/work environment	Retrospective longitudinal DID	Key predictor: Unemployment/work environment had nonsignificant effects
Ransome et al. (2020)^89^	New York City Neighborhood	2009–2014 Other acute cause	Economic context	Retrospective longitudinal Hierarchical Bayesian spatiotemporal multivariate Poisson regression model with multiple random effects	Key predictor: Economic disadvantage was a risk factor
Scherer et al. (2015)^35^	United States State	1982–2012 (pooled) DUI fatalities	Alcohol & drug policies	Cross-sectional (pooled data) Structural equation modeling	Key predictor: Alcohol & drug policies were a protective factor
Seto (2022)^74^	United States County	2010–2019 (pooled) Deaths of despair	Social context	Cross-sectional (pooled data) Spatial autoregressive models	Key predictor: Social context had mixed effects. Covariates: Racism/discrimination was a protective factor; built environment was a risk factor; and unemployment/work environment had mixed effects
Shiels et al. (2020)^65^	United States County	2000–2017 Other chronic cause	Built environment	Repeated cross-section Descriptive analysis; hot spot analysis for identifying clusters of high-rate counties	Key predictor: Built environment was a risk factor
Simon & Masters (2021)^75^	United States County	1990–2017 (pooled) Deaths of despair	Built environment	Cross-sectional (pooled data) Correlations in mortality change scores between 1990–1991 and 2016–2017 by urbanization	Key predictor: Built environment had nonsignificant effects
Singh & Siahpush (2014)^57^	United States County	1990–1992 & 2005–2009 Cirrhosis/liver disease	Built environment	Repeated cross-section Log-linear regression models; rate ratios or relative risks and rate differences comparing two time periods; Poisson regression models	Key predictor: Built environment was a risk factor
Singh & Siahpush (2016)^58^	United States County	1990–1992 & 2006–2010 Cirrhosis/liver disease	Employment/work environment	Repeated cross-section Descriptive analysis for 1990–1992 and 2006–2010 for low and high unemployment rates	Key predictor: Unemployment/work environment was a risk factor
Sloan et al. (1994)^81^	United States (excl. Alaska, Hawaii) State	1982–1988 Multiple	Alcohol & drug policies	Pre-test/post-test Fixed effects regression modeling, weighted by state population ages 25 to 64 years; some models removed time series variation using year indicator variables	Key predictor: Alcohol & drug policies had mixed effects
Son & Topyan (2011)^82^	United States State	1995–2004 Multiple	Alcohol & drug policies	Retrospective longitudinal/lagged Regression weighted by state-year population ages 25 to 64 years with 1-year lag from excise taxes	Key predictor: Alcohol & drug policies had mixed effects
Spencer et al. (2020)^70^	United States County	2000–2018 AOD combined	Built environment	Observational panel analysis Joinpoint weighted least-squares regression models	Key predictor: Built environment was a risk factor
Spillane et al. (2020)^5^	United States State	2000–2016 Unspecified alcohol-related mortality	Built environment	Repeated cross-section Descriptive trends	Key predictor: Built environment was a risk factor
Stringer (2018)^46^	United States County	1993–2015 DUI fatalities	Social context	Retrospective longitudinal Multilevel growth curve modeling (level 1 = repeated measures over time, level 2 = county, level 3 = state)	Key predictor: Alcohol & drug policies were a protective factor Covariates: Economic disadvantage and education were protective factors; racism/discrimination and built environment had mixed effects
Stringer (2019)^47^	48 U.S. states & District of Columbia (excl. Florida, Illinois) County	1985–2015 DUI fatalities	Alcohol & drug policies	Retrospective longitudinal/lagged Multilevel latent growth curve modeling with 1-year lag	Key predictor: Social context was a protective factor Covariates: Built environment had mixed effects
Studnicki et al. (2005)^59^	Florida County	2001 Cirrhosis/liver disease	Built environment	Cross-sectional Descriptive comparisons of selected health status indicators across populations, time	Key predictor: Built environment was a risk factor
Subbaraman et al. (2021)^66^	United States State	1999–2016 Other chronic cause	Alcohol & drug policies	Retrospective longitudinal/lagged Fixed-effect log-log models with 1-year lag for policies	Key predictor: Alcohol & drug policies had mixed effects
Teoh et al. (2021)^36^	United States (excl. California) State	2001–2019 DUI fatalities	Alcohol & drug policies	Repeated cross-section Poisson regression with log link scale parameter to allow for overdispersion	Key predictor: Alcohol & drug policies were a protective factor
Ullman (2016)^37^	United States (excl. Alaska, District of Columbia) State	2001–2012 DUI fatalities	Alcohol & drug policies	Retrospective longitudinal Weighted least-squares regression models; DID fixed effects	Key predictor: Alcohol & drug policies were a protective factor Covariates: Economic disadvantage was a protective factor; built environment was a risk factor; and racism/discrimination had mixed effects
Voas et al. (2000)^38^	United States State	1982–1997 (pooled) DUI fatalities	Alcohol & drug policies	Cross-sectional (pooled data) Weighted least-squares regression models	Key predictor: Alcohol & drug policies were a protective factor
Wagenaar et al. (2009)^67^	Alaska State	1976–2004 Other chronic cause	Alcohol & drug policies	Lagged time series analysis Combination of ARIMA model with structural parameters in interrupted time-series models	Key predictor: Alcohol & drug policies were a protective factor
Wolf et al. (2022)^90^	United States State	1999–2019 (pooled) Other acute cause	Employment/work environment	Cross-sectional (pooled data) Negative binomial regression with fixed effects for state and year and adjusted standard errors for state-level clustering; the predicted mortality using counterfactual values	Key predictor: Unemployment/work environment had mixed effects
Wright & Lee (2021)^48^	United States (excl. Alaska, Hawaii)	1986–2004 DUI fatalities	Alcohol & drug policies	Pre-test/post-test Longitudinal state fixed effects models; DID; and contiguous counties fixed effects designs	Key predictor: Alcohol & drug policies were a protective factor
Zeglin et al. (2019)^76^	Florida County	2016 Deaths of despair	Multiple	Cross-sectional Backwards regression	Key predictors: Economic disadvantage and education were protective factors; health care/social services and built environment had mixed effects
Zemore et al. (2022)^86^	Four U.S.-Mexico border states County	2008–2017 (pooled) Multiple	Built environment	Cross-sectional (pooled data) Spatial lag models	Key predictor: Built environment had mixed effects Covariates: Racism/discrimination had mixed effects; other predictors had nonsignificant effects
Zimmerman et al. (2021)^96^	United States State	1976–2015 Other acute cause	Health policies	Retrospective longitudinal Generalization of the quasi-experimental synthetic control method incorporating least absolute shrinkage and selection operator (LASSO)-penalized linear elastic net regression	Key predictor: Health policies were a protective factor

*Notes:* AOD, alcohol and other drugs; ARIMA, autoregressive integrated moving average; DID, difference-in-differences; DUI, driving under the influence; NVDRS, National Violent Death Reporting System. A spreadsheet with the detailed information is available upon request from the corresponding author.
